# Addressing the Gaps and Challenges of Autochthonous Mucocutaneous Leishmaniasis in Spain

**DOI:** 10.3390/tropicalmed11080235

**Published:** 2026-08-21

**Authors:** Diego Gayoso-Cantero, Begoña Monge-Maillo, Jose A. Perez-Molina

**Affiliations:** 1 National Referral Centre for Tropical Diseases, Infectious Diseases Department, University Hospital Universitario Ramón y Cajal, Instituto Ramón y Cajal de Investigación Sanitaria (IRYCIS), 28034 Madrid, Spain; begomongem@gmail.com (B.M.-M.); jperezm@salud.madrid.org (J.A.P.-M.); 2 Centro de Investigación Biomédica en Red Enfermedades Infecciosas (CIBERINFEC), Instituto de Salud Carlos III, 28029 Madrid, Spain; 3 World Health Organization Collaborative Centre for Clinical Management of Leishmaniasis (SPA-55), 28034 Madrid, Spain; 4 Health Sciences, University of Alcalá, 28871 Alcalá de Henares, Spain

**Keywords:** *Leishmania*, *Leishmania infantum*, mucocutaneous leishmaniasis, autochthonous, Spain

## Abstract

Autochthonous mucocutaneous leishmaniasis caused by *Leishmania infantum* is an under-recognised but established manifestation of endemic leishmaniasis in Spain. This review identified 65 confirmed autochthonous cases reported since 1990, showing a relatively consistent clinical pattern. Most patients were adults, predominantly men in the fifth or sixth decade of life. Nearly half were immunosuppressed, mainly due to HIV infection, corticosteroid use, biologic therapy, autoimmune diseases, or solid organ transplantation, although immunocompetent individuals were also affected. The laryngeal, nasal, and oral mucosa were the most commonly involved sites, with occasional tracheal and bronchial disease. Clinical presentation was typically chronic and non-specific, including ulcerative, nodular, polypoid, or tumour-like lesions that frequently mimicked malignancy or inflammatory disorders, often delaying diagnosis. Unlike classical New World mucocutaneous leishmaniasis, Spanish cases generally lacked a preceding cutaneous lesion, supporting recognition as a distinct form of *L. infantum* infection. Diagnosis required microbiological confirmation, ideally with species identification through culture or molecular methods, while serology showed limited reliability. Treatment approaches varied considerably, reflecting the absence of standardised protocols, although systemic therapy (particularly liposomal amphotericin B) was most commonly used. Improved recognition, surveillance, and inclusion in clinical guidelines are needed to reduce diagnostic delay and therapeutic variability.

## 1. Introduction

Leishmaniasis remains one of the most significant neglected tropical diseases transmitted by vectors. It is caused by more than 20 *Leishmania* species transmitted by phlebotomine sandflies, affecting nearly 350 million people in endemic regions worldwide. In 2022, the WHO officially reported 205,986 cases of cutaneous and 12,842 cases of visceral leishmaniasis, though underreporting is considerable, especially in Europe, where only 8–11% of endemic countries submitted data Ruiz [1]. The Mediterranean basin is the main area of local transmission in the WHO European Region, with *Leishmania infantum* the sole endemic species responsible for both visceral (VL) and cutaneous forms [1,2,3]. Spain continues to be one of the European countries with the highest number of locally acquired leishmaniasis cases, accounting for roughly 75% of the regional burden, along with Italy, Albania, and Georgia [3]. Spain continues to report one of the highest numbers of autochthonous leishmaniasis cases in Europe. In this context, it should be noted that surveillance systems and reporting requirements vary considerably across European countries, and no harmonised mandatory surveillance system exists at the European Union level. Consequently, the reported burden is influenced not only by the underlying epidemiology of *Leishmania infantum* infection but also by differences in case detection, notification, and national surveillance practices. Nevertheless, Spain represents a well-established endemic setting for *L. infantum*, with widespread distribution of competent sandfly vectors and animal reservoirs, together with relatively high clinical awareness and access to diagnostic techniques. These factors probably contribute both to the sustained occurrence of autochthonous cases and to their recognition and reporting in the scientific literature.

In the Old World, mucosal and mucocutaneous leishmaniasis (MCL) are considered uncommon entities, in contrast to the classical South American pattern of MCL associated with *L. braziliensis*, *L. panamensis,* and *L. guyanensis* [4,5]. However, cases of mucosal involvement caused by *L. infantum* have been increasingly recognised across southern Europe [6,7,8,9,10,11,12,13,14,15,16]. These forms are often misdiagnosed as malignancy or chronic granulomatous disease, especially when mucosal lesions occur without a preceding or concurrent cutaneous ulcer [7].

Clinically, mucosal involvement mainly affects adult males and immunocompromised individuals, particularly people living with HIV or those receiving corticosteroids or immunosuppressants [6,10,13,16,17]. Diagnostic delays are common, and the disease often requires systemic therapy with liposomal amphotericin B, miltefosine, or pentavalent antimonials [5,18,19]. However, current international guidelines rarely address autochthonous Old World MCL, focusing primarily on imported cases from Latin America [2].

This review offers an updated overview of mucocutaneous leishmaniasis in Spain caused by *Leishmania infantum*, emphasising its epidemiology, clinical features, diagnosis, and treatment, while highlighting ongoing gaps in recognition and surveillance within the European context.

## 2. Material and Methods

A literature search was performed in PubMed and Embase from database inception to February 2026 to identify published cases of human mucocutaneous leishmaniasis acquired in Spain. The search terms included “leishmaniasis”, “mucocutaneous leishmaniasis”, “MCL”, and “Spain”, combined as appropriate for each database. Eligible reports were those describing human mucocutaneous leishmaniasis caused by *Leishmania infantum*, with species confirmation and evidence supporting acquisition in Spain. Reports were excluded if they described exclusively cutaneous or visceral leishmaniasis, cases caused by other *Leishmania* species, cases probably acquired outside Spain, non-human infections, reviews without original case-level data, or duplicate reports. A secondary search was also performed by reviewing the reference lists of included articles and other potentially relevant publications. Study selection and data extraction were performed using a predefined set of clinical, epidemiological, diagnostic, and therapeutic variables; extracted data were subsequently checked for consistency before analysis.

Titles, abstracts and full texts were screened independently by two reviewers, and data extraction was performed using a predefined set of clinical, epidemiological, diagnostic, and therapeutic variables. Discrepancies were resolved by consensus.

## 3. Relevant Sections

### 3.1. Epidemiology

Cutaneous leishmaniasis (CL) has been recognized in Spain since the early 20th century, with initial descriptions along the Mediterranean coast. It subsequently became established as an endemic condition, particularly in rural areas of the eastern and southern regions [20]. *Leishmania infantum* is the sole aetiological agent, and current estimates indicate an incidence of 0.41 cases per 100,000 inhabitants [21]. Canine infection remains widespread, with seroprevalence ranging from 4% to 35% across regions and 7.8% in Madrid, where many infected dogs show no clinical signs [21,22]. The main vectors involved in transmission are *Phlebotomus perniciosus* and *P. ariasi* [22].

The number of autochthonous cases has shown a slight increase from 2016 to 2019 (incidence rate = 0.78 to 0.90 cases per 100,000 inhabitants, respectively), followed by a decline in 2020 (IR 0.58 per 100,000 inhabitants) and a moderate resurgence up to 2023, reaching an IR of 0.80 per 100,000 inhabitants [23]. In 2023, a total of 394 cases were reported. Clinical information was available for 351 of them: 187 (52.2%) were visceral leishmaniasis (VL), and 171 (47.8%) were cutaneous/mucosal forms. The least frequent category was MCL (*n* = 7), accounting for only 2.0% of all reported cases [23]. However, despite its long-standing presence, leishmaniasis remains markedly underreported in Spain. Humanes-Navarro et al. estimated underreporting rates of 17.6% for VL, 53.9% for CL, and up to 70% for MCL, highlighting substantial deficiencies in national surveillance [3].

Similarly, in a European multicentre study of travellers with leishmaniasis, Spain was identified as the country of origin for the highest number of tegumentary leishmaniasis cases (*n* = 48) [14]. Among the 166 cases with identified *Leishmania* species, eight cases were mucocutaneous leishmaniasis (MCL) and seven mucosal leishmaniasis, all acquired in the Old World and caused by *Leishmania infantum*. Seven of these cases were contracted in Spain, with the remaining originating from France (2), Italy (2), Greece (2), Oman (1), and Turkey (1), demonstrating Spain’s importance as a major focus within the Mediterranean basin.

Mucosal involvement in leishmaniasis is probably multifactorial and results from the interaction between parasite-, host- and environment-related factors. In the New World, mucosal disease is classically associated with species of the *Leishmania* (*Viannia*) subgenus, particularly L. braziliensis, although other species such as *L. guyanensis* and *L. panamensis* have also been implicated [24]. Parasite dissemination from the primary cutaneous lesion, parasite burden, strain-specific virulence factors and the ability to persist in tissue may contribute to mucosal tropism. In addition, the presence of *Leishmania* RNA virus, mainly described in *L.* (*Viannia*) species, has been associated with increased inflammatory responses and more severe mucocutaneous disease [25]. From the host perspective, mucosal leishmaniasis is characterised by an intense and poorly regulated cell-mediated immune response, with increased production of pro-inflammatory cytokines such as IFN-γ and TNF-α and insufficient counter-regulation by IL-10 and TGF-β, leading to chronic inflammation and tissue destruction despite low parasite density in lesions [26,27]. Geographical factors are also relevant, as circulating *Leishmania* species, vectors, reservoirs and host genetic background vary across endemic areas and influence clinical phenotypes [24,28]. In the Mediterranean basin, mucosal involvement due to *L. infantum* has been reported but remains uncommon, and the mechanisms underlying this presentation are less well defined than those described for American tegumentary leishmaniasis [16].

### 3.2. Clinical Features

A review of published series and case reports identified 65 confirmed cases caused by *L. infantum* since 1990 and acquired in endemic regions of Spain (Table 1). MCL due to *L. infantum* has been reported mainly in adults, with a clear male predominance and a median age around the fifth to sixth decade of life. Among the 65 documented cases, nearly half occurred in immunosuppressed individuals, most frequently in the context of HIV infection, systemic corticosteroid use, autoimmune disease treated with biologics, or solid organ transplantation. The most commonly affected mucosal sites were the larynx, nasal, and oral mucosa, although tracheal and bronchial involvement was also described. Clinical manifestations included ulcerative, nodular, or polypoid lesions, often leading to dysphonia, nasal obstruction, odynophagia, or dyspnea, and in several cases mimicking neoplastic or inflammatory diseases. Delayed diagnosis was common, with chronic evolution over several months. Although most patients were immunocompromised, mucosal leishmaniasis also occurred in immunocompetent individuals, highlighting the expanding clinical spectrum of autochthonous *L. infantum* infection in Spain.

**Table 1:** Main clinical characteristics of cases of *Leishmania infantum* mucocutaneous leishmaniasis acquired in Spain.

Reference	No. of ML Cases	Age(y)/Sex	Immunosuppression (*n*, %)	Main Mucosal Sites	Clinical Features
Alvar et al. 1990 [29]	1	62/M	No	Nasal mucosa	Right nasal vegetative firm tumor involving the cartilaginous right septum
Cortés et al. 1997 [30]	1 (two episodes)	30/M	HIV infection	1. Oral cavity 2. Oral cavity	1. Vegetative tumour-like ulcer in the left region of the hard and soft palate 2. Ulcerous sublingual lesions
Aliaga et al. 2003 [6]	31 (14 Spain)	Mean 51, 27M/4F	14/31 (45%)—HIV, steroids, transplant	Larynx (13), oral (6), nasal (5), combined (7)	Ulcerative/nodular lesions; dysphonia, nasal obstruction, odynophagia; chronic evolution (mean 12 months)
García de Marcos et al. 2007 [12]	3	70 M, 41 M, 50 M	2/3 (67%)—HIV infection	Oral mucosa (3)	Painful ulcerated oral lesions (floor of mouth, cheek mucosa, gingiva); chronic course
Cobo et al. 2016 [7]	6	36–67; 4M/2F	3/6 (50%)—HIV, RA (biologics/steroids), diabetes/asthma	Nasal (3), larynx (3)	Polypoid or tumor-like lesions; dysphonia, nasal obstruction, odynophagia; often mistaken for cancer
Patel et al. 2017 [16]	3 (imported Old World ML)	72 M, 69 M, 60 F	3/3 (100%)—local immunosuppression and corticosteroids	Nasal (1), vocal cord (2), trachea (1)	Imported to UK: 3/5 acquired in Spain. Addison disease (1); RA (1); Asthma (1)
Garrido-Jareño et al. 2020 [13]	5 (of 42 total)	Adults/NR	3/5 (60%)—immunosuppressed (1 biologic)	Nasal (2), oral (3)	Ulcerative/purulent lesions; diagnosis delay; In all cases, the initial diagnosis was epidermoid carcinoma,
Guery et al. 2021 [14]	7 (Multicentre European series)	—	—	Nasal, oral and laryngeal	50% immunosuppressed; middle aged adults; diagnosis delay 7 months
García-Callejo et al. 2021 [11]	4 (mucosal subset of 13 ENT cases)	53–68; 3F/1M	1/4 (25%)–RA (biologic)	Nasal (3), vocal cord (1)	Chronic rhinolaryngeal lesions with dysphonia/obstruction; diagnostic delay (4–7 months)
Lores Benavente et al. 2025 [15]	3	68 M, 39 M	0/3 (0%). All immunocompetent (1 started systemic steroids)	Trachea/bronchi (1), laryngeal (2)	Dyspnea, dysphonia, laryngeal edema, airway obstruction; nodular/edematous mucosa; recurrent course in 1 case
Eggers et al. 2025 [8]	1	77 M	No	Tongue/oral mucosa	Ulcerous lesion; suspected reactivation from a previous CL

CL: cutaneous leishmaniasis; F: female; M: male; ML: mucosal leishmaniasis; NR: not reported; RA: rheumatoid arthritis.

### 3.3. Diagnosis

Diagnosis of MCL generally relies on a combination of clinical and epidemiological suspicion alongside laboratory confirmation, since mucosal manifestations are nonspecific and may resemble other granulomatous or ulcerative conditions. In the reported series, sampling was performed preferentially from visibly abnormal mucosa, most often nasal, oral, or pharyngeal sites, using biopsy specimens that allowed simultaneous histopathological assessment (including exclusion of alternative diagnoses) and molecular testing.

Direct parasitological confirmation by microscopy of Giemsa-stained smears, imprint preparations, or tissue sections was used as a highly specific method, although sensitivity was variable and often limited in mucosal disease due to low parasite burden, lesion characteristics, and technical expertise [2,2,24].

Fine-needle aspiration cytology and rapid imprint-based approaches, such as press–imprint–smear, were described as strategies to improve yield, and immunohistochemistry was used in selected cases to enhance visualisation of amastigotes when organisms were scarce. Immunohistochemical staining (e.g., with anti-CD1a) may further improve the detection of amastigotes [8].

Culture provides definitive parasitological confirmation and potential downstream typing, but is less frequently favoured because it is technically demanding, prone to contamination, and slow, particularly in low-density lesions. Cultures may become positive within a week in parasite-rich lesions but can take up to 30 days when parasite density is low. Histopathology supports diagnosis and staging and commonly shows granulomatous inflammation with detectable parasites. Overall, histopathology supports diagnosis and staging but should be complemented by parasitological or molecular techniques for confirmation [31].

DNA-based assays (PCR and, where available, quantitative PCR) were the most sensitive tools, applicable to mucosal biopsy material and also to less invasive lesion samples such as smears or swabs in some reports, and additionally enabled species identification through PCR-based typing approaches (e.g., RFLP or sequencing), which was considered relevant for clinical management and epidemiological surveillance [31]. Molecular methods not only increase diagnostic sensitivity but also enable precise species identification, and have been proven superior to traditional microscopy or culture in terms of sensitivity, particularly when combined with less invasive sampling methods. Quantitative PCR (qPCR) additionally enables parasite load estimation and may be useful in monitoring treatment response or predicting therapeutic failure [32]. In Spain, PCR-based molecular identification of *Leishmania* species is available in many centres and through the National Centre for Microbiology, which acts as the national reference laboratory. Although species-specific PCR may not be performed locally in every hospital, clinical samples can be referred to this reference centre, allowing access to molecular confirmation throughout the country when clinically indicated.

Molecular approaches have significantly enhanced leishmaniasis diagnostics in Spain, where studies analysing clinical isolates from 1981 to 2018 demonstrated increasing diversity and incidence, particularly among imported cases. Combining molecular and enzymatic methods allowed identification of multiple strains, while MALDI-TOF and targeted gene sequencing provide accurate, clinically practical tools for species-level discrimination.

Serological assays were generally of limited value in MCL because antibody titres are often low or absent, with potential utility mainly in advanced mucosal disease or as an adjunct to monitoring severity and treatment response, recognising possible cross-reactivity [33]. In general, higher levels of antibodies have been reported for ML-patients compared to CL-patients, the latter being characterized by a moderate Th1 immune response [34].

In the cases included in our review, biopsies for histological and microbiological examination, including Giemsa staining, were frequently performed, mainly because of lesion location and diagnostic uncertainty. In some cases, these procedures were complemented by culture in NNN medium or PCR-based techniques. Among serological methods, indirect immunofluorescence antibody testing (IFAT/IFI) is notable, as it often yielded negative results despite confirmed infection, although positive cases tended to show high antibody titres. Direct agglutination tests (DAT) and rK39-based assays were also used to detect anti-*Leishmania* antibodies. All cases included in our study had confirmed *Leishmania infantum* infection, demonstrated by either culture or PCR (Table 2).

**Figure 1:**
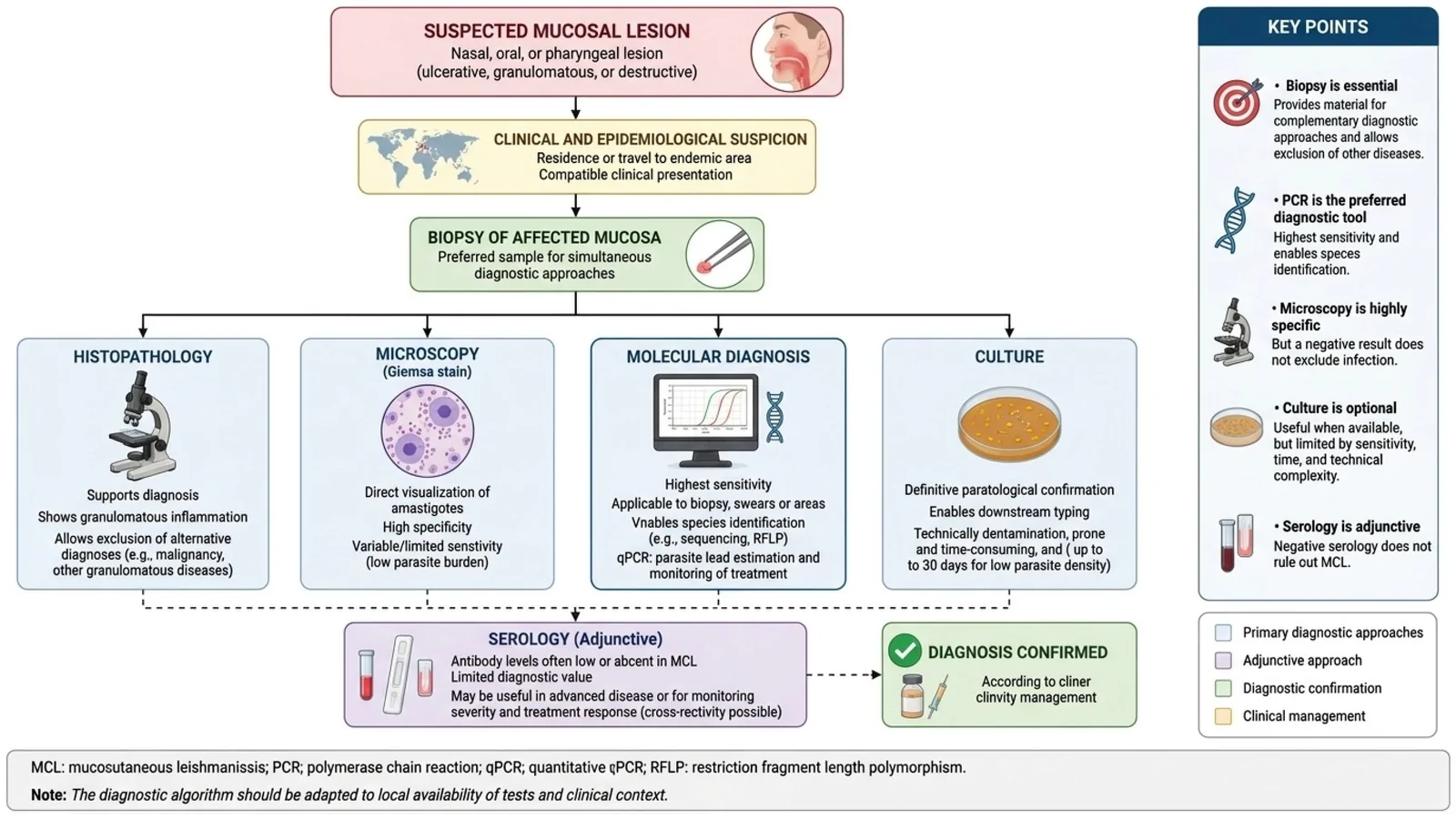
Recommended diagnosis algorithm.

The main diagnostic methods used for MCL are summarized in Figure 1.

**Table 2:** Main diagnostic approaches and treatments employed in cases of *Leishmania infantum* mucocutaneous leishmaniasis acquired in Spain.

Reference	Diagnostic Methods	Parasitological Treatment
Alvar et al. 1990 [29]	A biopsy was performed with successful isolation of the parasite in NNN medium. Molecular characterization by in situ hybridization, using a nuclear DNA probe derived from a cDNA library of *Leishmania* (*L.*) *infantum* (strain MHOM/MA/67/ITMAP-264), showed a strong signal in both nuclear and kinetoplast DNA, identifying the isolate as *L.* (*L.*) *infantum*. This identification was confirmed by isoenzyme analysis, whose electrophoretic profile corresponded to *L.* (*L.*) *infantum* variant 1 (zymodeme MON-1; LLM-107). Immunologically, no inversion of the CD4/CD8 (OKT4/OKT8) lymphocyte ratio was observed, and specific anti-*Leishmania* serology (IFAT, EIA, and counterimmunoelectrophoresis) yielded borderline results.	Meglumine antimoniate (Glucantime^®^) (20 mg pentavalent antimony/kg/day) for 20 days.
Cortés et al. 1997 [30]	**First lesion:** Biopsy samples were taken, leading to a diagnosis of leishmaniasis by microscopical examination and growth of promastigotes in Now-McNeal-Nicolle’s I medium (NNN). Isoenzyme analysis of the strain was carried out according to the Montpellier technique, and the strain (MHOM ES/94/BCN113) was identified as *L. infantum* MON-1. Specific humoral immune response to *L. infantum* antigens was detected by the immunofluorescence antibody test (IFAT), with a titre of 1/2560. The immunoblot was also positive, with the characteristic band pattern of *L. infantum.* **Second lesion**: Biopsy of the lesion revealed parasites *Leishmania* bv by microscopical examination and bv culture in NNN medium. The strain (MHOM/ES/95/BCN137) was identified by isoenzyme typing as L. infantum M6N-34. *Leishmania* serology by IFAT was again positive, with a titre of l/160.	**First lesion**: intramuscular Meglumine antimoniate (Glucantime^®^) (20 mg pentavalent antimony/kg/day) for 20 days and oral allopurinol 500 mg twice a day for 20 days. **Second lesion**: oral itraconazole, 100 mg twice a day, and allopurinol, 5 mg/kg every 6 h for 4 weeks.
Aliaga et al. 2003 [6]	**Patient 1**: A biopsy was performed, and histologic examination revealed *Leishmania* spp. amastigotes. Culture of the biopsy was performed, which was also positive for *Leishmania*, identified as *L.* (*L.*) *infantum* zymodeme MON-24. Bone marrow aspiration for parasite examination and culture was negative. Indirect immunofluorescent assay (IFA) for antileishmanial antibodies was negative.	Meglumine antimoniate (Glucantime^®^) (20 mg pentavalent antimony/kg/day) for 28 days.
	**Patient 2**: Biopsy of the oral lesions revealed amastigotes of *Leishmania* spp. Tissue culture grew *Leishmania*, identified as *L.* (*L.*) *infantum* MON-183 var NP1. Bone marrow aspiration for parasite examination and culture was negative. Indirect immunofluorescent assay (IFA) was positive at a titer of 1:80.	Amphotericin B lipid complex (150 mg/day), discontinued after 2 doses due to dyspnea. Meglumine antimoniate (Glucantime^®^) (20 mg/kg/day) was initiated but discontinued on day 3 due to acute pancreatitis. After recovery, itraconazole (200 mg/day) and allopurinol (300 mg/day) were administered for 75 days.
	**Patient 3**: Pharyngeal biopsy showed granulomatous inflammatory reaction with intracellular *Leishmania* spp. within macrophages. Culture grew *Leishmania* spp. species, identified as *L.* (*L.*) *infantum*. Esophageal biopsies and bone marrow aspiration for examination and culture were negative. Indirect immunofluorescent assay (IFA) was positive at a titre of 1:80.	Meglumine antimoniate (Glucantime^®^) (20 mg/kg/day) for 28 days.
García de Marcos et al. 2007 [12]	**Patient 1**: Biopsy of the lesion was obtained. Histology showed epithelium with chronic inflammatory infiltrate predominantly composed of plasma cells and histiocytes, with cytoplasm invaded by *Leishmania* spp. parasites. Bone marrow biopsy showed no alterations. Immunofluorescence revealed a *Leishmania* IgG antibody titer of 1:320.	Meglumine antimoniate (Glucantime^®^) 20 mg/kg/day for 28 days.
	**Patient 2**: Histopathological study of the biopsy revealed leishmaniasis, but no further information was provided.	Meglumine antimoniate (Glucantime^®^) 20 mg/kg/day; subsequently withdrawn. Total duration of treatment with meglumine antimoniate was one month. Secondary prophylaxis with pentamidine isethionate was initiated at 4 mg/kg body weight every 15 days and was maintained at the time of the last control; however, the interval until this last follow-up was not specified.
	**Patient 3**: Oral mucosal biopsy showed numerous macrophages containing abundant *Leishmania* amastigotes, while the overlying epithelium appeared preserved without significant alterations. PAS (Periodic Acid–Schiff) staining highlighted intensely stained intracellular *Leishmania* parasites within the cytoplasm of macrophages.	No treatment (lost to follow-up).
Cobo et al. 2016 [7]	**Patient 1**: Histological examination of the lesion biopsy revealed a non-specific inflammatory infiltrate with *Leishmania* spp. amastigotes in the tissue. Culture of the biopsy specimen was positive for *Leishmania*. PCR for *L. infantum* was positive.	Liposomal amphotericin B (300 mg/day) for 9 days.
	**Patient 2**: Histological examination of the lesion biopsy revealed a non-specific inflammatory infiltrate with *Leishmania* spp. amastigotes in the tissue. Culture of the biopsy specimen was positive for *Leishmania*. PCR for *L. infantum* was positive	Liposomal amphotericin B (300 mg/day) for 9 days.
	**Patient 3**: Histological examination of three biopsies showed a granulomatous inflammatory reaction without visualization of amastigotes. Culture from a new biopsy grew *Leishmania* promastigotes. Nested PCR was positive for *L. infantum*.	Liposomal amphotericin B (300 mg/day), discontinued due to arterial hypertension and lumbar pain; subsequently treated with meglumine antimoniate (Glucantime^®^) (20 mg/kg/day). The duration until discontinuation of the first treatment and the duration of the second treatment were not specified.
	**Patient 4**: Biopsies of the oral lesions showed a granulomatous chronic inflammatory infiltrate. Giemsa staining revealed no intracellular amastigotes. A new biopsy was obtained; nested PCR was positive for *L infantum*, and culture grew *Leishmania* promastigotes. Anti-*Leishmania* antibodies were detected at a titer of 1:80 by indirect immunofluorescence assay. PCR for *L. infantum* was positive.	Liposomal amphotericin B (200 mg/week) for 5 weeks with no improvement; new cycle with liposomal amphotericin B (300 mg/day) achieved complete resolution of lesions. The duration of the second cycle was not specified.
	**Patient 5**: Biopsy of the lesions showed a granulomatous inflammatory infiltrate with Giemsa-stained *Leishmania* amastigotes. Culture of the biopsy specimen was positive for *Leishmania* promastigotes. PCR for *L. infantum* was positive.	Liposomal amphotericin B (300 mg/day) for 5 weeks.
	**Patient 6**: Direct examination of a Giemsa-stained sample showed abundant *Leishmania* amastigotes; culture was positive for *Leishmania*. PCR for *L. infantum* was positive. A granulomatous inflammatory infiltrate was observed. Indirect immunofluorescence assay for *Leishmania* was negative. Bone marrow aspiration for parasite examination was negative.	Meglumine antimoniate (850 mg/day Sb) for 28 days; lesions resolved after treatment.
Patel et al. 2017 [16]	**Patient 1**: a biopsy was taken of the local tissue and this demonstrated evidence of *Leishmania* amastigotes and was confirmed with detection of *L. donovani* complex DNA by polymerase chain reaction (PCR).	30-day course of oral miltefosine, 150 mg once daily.
	**Patient 2:** Histology of a total excision biopsy demonstrated numerous *Leishmania* amastigotes, confirmed with detection of *L. donovani* complex DNA by PCR. Leishmania direct agglutination test (DAT) was positive at a low titer of one in 3200.	A 30-day course of miltefosine was started. Dose not specified. In the absence of specific information, the same dosing as in patient 1 (150 mg once daily) was assumed.
	**Patient 3**: A biopsy detected *L. donovani* complex DNA by PCR. A *Leishmania* DAT was negative but the K39 antibody was positive	Once daily intravenous sodium stibogluconate (SSG) for 28 days. Dose not specified. In the absence of specific information, the same dosing as in patient 1 (150 mg once daily) was assumed.
	**Patient 4**: Histology demonstrated numerous amastigotes of *Leishmania*, confirmed with detection of L. donovani complex DNA by PCR. Leishmania DAT was positive at a titer of 1 in 102,400 and K39 antibody was positive.	30-day course of miltefosine. In the absence of specific information, the same dosing as in patient 1 (150 mg once daily) was assumed.
	**Patient 5**: Histology of a biopsy demonstrated amastigotes of *Leishmania*, confirmed with detection of *L. donovani* complex DNA by PCR. *Leishmania* DAT was negative and K39 antibody weakly positive.	30-day course of miltefosine. Dose not specified. In the absence of specific information, the same dosing as in patient 1 (150 mg once daily) was assumed.
Garrido-Jareño et al. 2020 [13]	Patients with cutaneous leishmaniasis (CL) *n* = 37 and mucocutaneous leishmaniasis (MCL) *n* = 5 are not described separately in the paper. Giemsa-stained lesion smears: performed in 18 patients; positive in 13/18. Histopathological study: performed in 36 patients; amastigotes identified in 20/36. NAAT (nucleic acid amplification tests): performed in 36 patients; positive in 33/36. *Leishmania infantum* in 32 cases. NAAT-negative cases: 3 patients—diagnosis by histology in 2, and by combination of smear + histology in 1. Cases diagnosed only by NAAT: 13/42, because amastigotes were not visualized by microscopy. Patients without NAAT: 6—diagnosis confirmed by Giemsa stain.	Focusing on MCL cases (*n* = 5), the majority of patients were treated with amphotericin B in monotherapy (4/5) and in the remaining patient recovery occurred after the diagnostic biopsy.
Guery et al. 2021 [14]	15 patients from the Old World: 7 had ML and 8 had MCL. Thirty-six patients had *L. infantum* infection, of whom 22% had cutaneous involvement.	The treatments received by patients with mucosal involvement due to *L. infantum* were not individually specified. The treatments that were used included: antimonial therapy (MA or SSG), Amphotericin B (liposomal or deoxycholate), Fluconazole, Miltefosine, Pentamidine Isethionate, Other: SSG + pentoxifylline (*n* = 1).
García-Callejo et al. 2021 [11]	In 12 patients, the diagnosis was made from smears of exudates or tissue biopsies of the affected area, with amastigotes of the protozoan identified inside histiocytes. These were easily recognized by their staining affinity with Giemsa, although intracellular protozoa could also be detected with hematoxylin-eosin. Since 2007, diagnosis has been confirmed by detection of kinetoplast DNA using polymerase chain reaction (PCR).	**Patient 1**: intravenous antimony (Sb IV) combined with excision of the lesion. **Patient 2**: excision along with intralesional antimony (Sb IL). **Patient 3**: excision and intravenous antimony (Sb IV). **Patient 4**: intravenous amphotericin B (AFB IV).
Lores Benavente et al. 2025 [15]	**Patient 1**: *Leishmania* DNA was detected by PCR in a laryngeal biopsy.	Initially received liposomal amphotericin B (40 mg/kg total over one month). After a relapse, the patient received weekly liposomal amphotericin B (4 mg/kg) for one month, followed by monthly maintenance for one year.
	**Patient 2**: Histology confirmed tracheal and bronchial leishmaniasis	Treated with liposomal amphotericin B.
	**Patient 3**: Laryngeal and tracheal biopsies identified intracellular microorganisms consistent with *Leishmania* spp.	Received five days of liposomal amphotericin B during hospitalization, followed by monthly doses.
Eggers et al. 2025 [8]	Initial tissue smear was negative by microscopy and routine PCR, but IFAT detected anti-*Leishmania* antibodies (1:1280). Kinetoplast PCR from the smear was positive, though insufficient for sequencing. Formalin-Fixed Paraffin-Embedded (FFPE) tissue histology revealed amastigotes, and PCR/sequencing identified *Leishmania infantum*. At follow-up, a new tongue lesion was confirmed as *L. infantum* by PCR and sequencing.	Treatment for the first lesion was initiated with oral fluconazole 200 mg/day for six weeks. A second lesion was treated with oral miltefosine 150 mg/day for 28 days.

### 3.4. Treatment of Mucocutaneous Leishmaniasis (MCL)

The therapeutic approach to MCL is fundamentally aimed at preventing morbidity and mortality associated with this condition. Treatment of MCL remains heterogeneous because standardised regimens are lacking and most evidence comes from uncontrolled reports, small series, and extrapolation from other forms of leishmaniasis. WHO guidance for Europe largely addresses mucosal disease in the context of imported species, leaving clinicians to adapt strategies for endemic *L. infantum* [2].

Published *L. infantum* MCL cases in Europe describe widely variable approaches, including local measures (surgery, intralesional antimonials, paromomycin, cryotherapy) for selected, limited lesions and diverse systemic regimens with varying drugs and cumulative doses [6,32,33,34,35].

Despite this variability, there is broad agreement that mucosal involvement generally warrants systemic therapy to prevent destructive sequelae and, in advanced pharyngo-laryngeal disease, potentially fatal complications. In significant laryngeal/pharyngeal involvement, hospitalization and adjunct corticosteroids are often recommended to mitigate treatment-triggered inflammatory worsening and airway compromise [2].

Liposomal amphotericin B (L-AmB) is a key option because of improved tolerability versus deoxycholate amphotericin B [36,37], but dosing is highly inconsistent across reports (approximately 18–60 mg/kg total over variable schedules), reflecting the absence of definitive dose–response data; failures and relapses are more frequent in immunocompromised patients, who may require higher cumulative doses and careful management of immunosuppression, and combinations (e.g., with miltefosine or immunomodulators) have been used only in isolated cases [6]. Observational data suggest *L. infantum* may respond particularly well to L-AmB compared with other species, and some authors propose adapting visceral leishmaniasis schedules with extended dosing until cure or a cumulative target (e.g., ~40 mg/kg) [37,38]. European guidelines recommend a total of 18–21 mg/kg administered on days 1–5 and day 10, 3–5 mg/Kg/Day [2]. This heterogeneity reflects the absence of definitive studies and underscores the need for more standardised protocols. In refractory cases, combinations of L-AmB with miltefosine, allopurinol, or subcutaneous interferon-γ have been used, although only in isolated case reports [39].

Miltefosine is the main oral systemic alternative with reported efficacy in Old World mucosal disease, although evidence remains limited [40,41]. Miltefosine may represent a valuable option for systemic oral treatment in immunocompetent patients with complicated OWCL and OWML, including cases of cutaneous involvement caused by *L. infantum*.

Pentavalent antimonials continue to be used but require close toxicity monitoring and are constrained by comorbidities and concerns about resistance. Meglumine antimoniate and sodium stibogluconate are the most widely used formulations, with WHO recommendations indicating a dosage of 20 mg of pentavalent antimony per kilogram per day for 30 days in mucosal disease, following a gradual dose escalation during the first days [2]. The toxicities necessitate close monitoring and limit their use in patients with cardiac, hepatic, or renal comorbidities [7].

Pentamidine is an additional option with significant toxicity and more established utility in American disease. Although treatment is relatively rapid and potentially less costly than antimonials, toxicity (including hypertension, tachycardia, gastrointestinal disturbances, elevation of muscle enzymes, renal failure, and pancreatic toxicity) limits its use as first-line therapy.

In the cases reported in our review, meglumine antimoniate was the most commonly used agent in earlier publications, typically at a dose of 20 mg of pentavalent antimony/kg/day for 28 days. Overall, however, liposomal amphotericin B appears to be the most frequently used treatment across all cases, with a clear increase in its use in more recent reports. Nevertheless, substantial heterogeneity remains, not so much in daily dosing, which was commonly 300 mg/day, but in treatment duration, which varied widely from 9 days to 5 weeks (Table 2). Other, less frequently used agents included miltefosine, fluconazole and combination regimens such as itraconazole with allopurinol. Pentamidine, in contrast, was reserved for secondary prophylaxis rather than for first-line treatment (Table 2).

**Figure 2:**
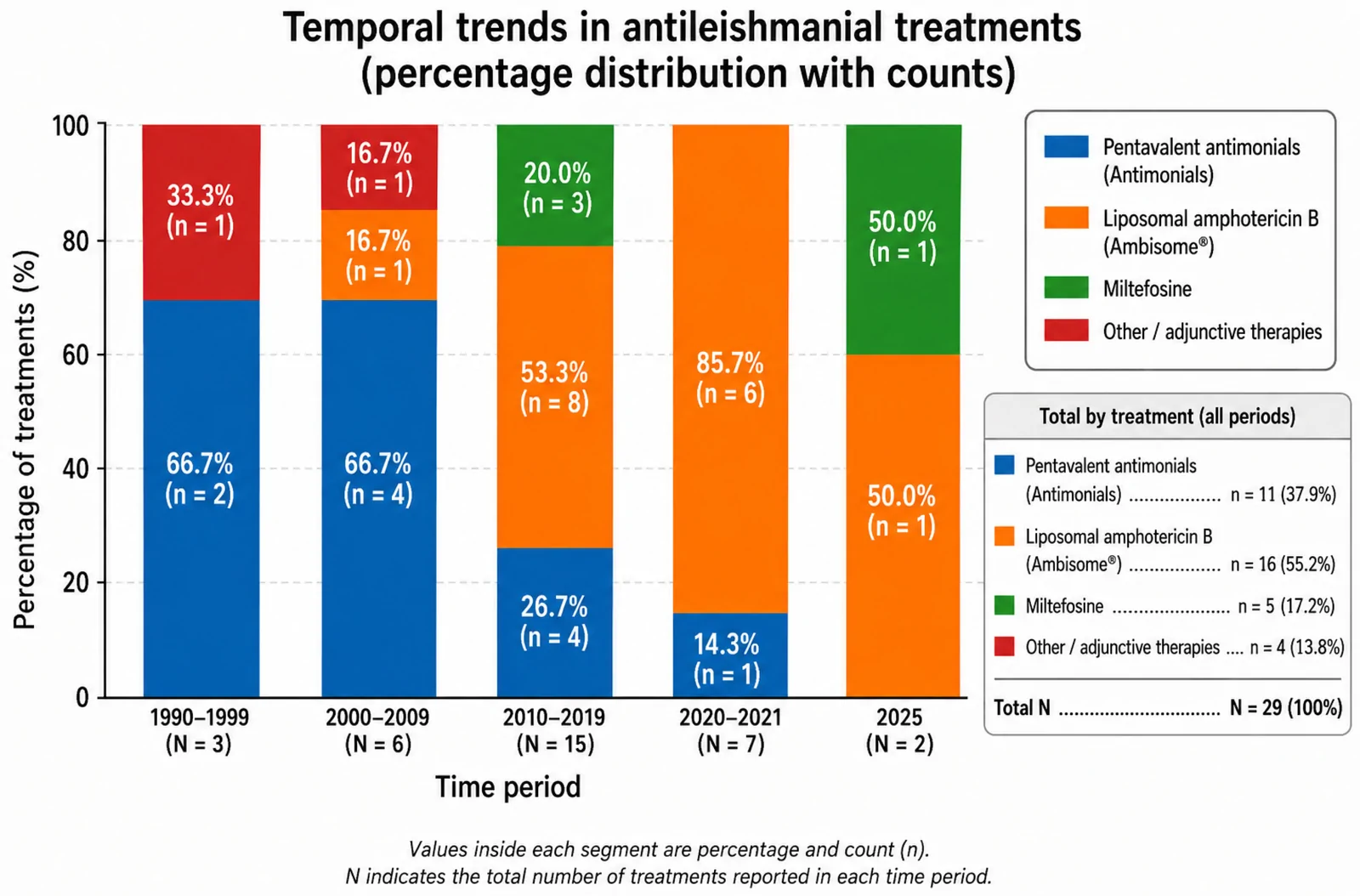
Temporal trends in the treatment of MCL caused by *L. infantum.* The temporal trends observed in treatment should be interpreted with caution. As this review is based exclusively on published case reports, publication and reporting bias may have influenced the apparent evolution of therapeutic strategies, and the patterns observed may not accurately reflect changes in routine clinical practice. In addition, individual authors and research groups frequently reported similar treatment approaches across different cases, suggesting that local expertise and physician preference may also have contributed to the observed heterogeneity. Nevertheless, the progressive shift from pentavalent antimonials to liposomal amphotericin B broadly parallels the evolution of therapeutic recommendations for Mediterranean visceral leishmaniasis caused by *Leishmania infantum*. During the 1990s, the increasing clinical experience with liposomal amphotericin B, driven by its improved safety profile, progressively changed clinical practice, and subsequent WHO, international, and Spanish guidelines established it as the preferred first-line treatment for Mediterranean visceral leishmaniasis. Although these developments may also have influenced the management of mucocutaneous disease, our study was not designed to evaluate this relationship, and no causal inferences can be drawn.

To illustrate changes in treatment trends over time, the treatments reported in the included studies across the study period are presented in Figure 2.

## 4. Discussion

Our review shows that autochthonous mucocutaneous leishmaniasis in Spain is not a rare manifestation of *Leishmania infantum* infection but rather a well-established yet under-recognised clinical condition. Published cases outline a fairly consistent profile, primarily affecting adults, often men and immunosuppressed individuals, but also including immunocompetent patients, thereby broadening the traditional view of those at risk. The disease most frequently involves the nasal, oral, and laryngeal mucosa, although cases involving the upper airway and tracheobronchial regions have also been reported. Its presentation is usually chronic and non-specific. Recognition is further complicated by surveillance systems that underrepresent mucocutaneous forms, despite evidence of underreporting. Additionally, treatment strategies across reported cases have varied considerably, with different drugs used in different treatment regimens and, very often, surgery. Overall, these findings underline the importance of maintaining a high suspicion for mucocutaneous leishmaniasis in Spain among patients with persistent mucosal lesions, while emphasising the need for more precise epidemiological recording and a more standardised therapeutic approach. Although autochthonous mucocutaneous leishmaniasis in Spain shares some features with classical New World mucocutaneous leishmaniasis, it seems to follow a distinct clinical pattern. Unlike New World mucocutaneous leishmaniasis, which usually affects otherwise immunocompetent patients, Spanish autochthonous disease due to *L. infantum* is concentrated in older adults, predominantly men, and nearly half have underlying immunosuppression, especially HIV infection, corticosteroid exposure, transplantation, or biologic therapy. Pathogenesis of isolated mucosal involvement in immunocompetent individuals remains poorly understood and represents a significant area for future research.

The geographical heterogeneity observed across Spain likely reflects regional differences in the eco-epidemiology of *Leishmania infantum*, including the distribution of competent sandfly vectors, the presence and density of animal reservoirs, and the complex interactions that maintain parasite transmission. Consequently, the risk of autochthonous infection is not evenly distributed throughout the country.

HIV infection was not uncommon among earlier reported cases, reflecting a period of more profound immunosuppression and poorer virological and immunological control. In recent years, however, the patient profile has shifted, with classical HIV/AIDS cases increasingly replaced by individuals with other forms of immunosuppression, as well as by immunocompetent patients. This broader spectrum is likely related to the expanding use of immunosuppressive therapies and advances in modern medicine, particularly solid organ transplantation. Although some agents, such as anti-TNF-α therapies, directly impair immune pathways involved in *Leishmania* control, similar presentations have also been reported with other immunosuppressive drugs not classically linked to impaired parasite control, suggesting that disease pathogenesis remains only partly understood. In addition, it is also important to distinguish mucocutaneous leishmaniasis caused by *L. infantum* from mucosal involvement occurring in the context of visceral leishmaniasis, such as gastrointestinal lesions. In the cases reviewed, mucocutaneous disease appears to represent a primary manifestation, often at sites distant from the presumed sandfly inoculation site, suggesting parasite dissemination without concomitant visceral involvement and supporting its recognition as a distinct clinical presentation.

In the Americas, mucocutaneous disease is usually associated with *Leishmania* (*Viannia*) species, especially *L. braziliensis*, and often develops months to years after a previous cutaneous lesion, frequently leaving residual scars [42]. In contrast, the cases acquired in Spain examined here most often presented as primary, isolated mucocutaneous disease without an identifiable preceding cutaneous lesion, thereby reducing clinical suspicion and delaying diagnosis. The anatomical distribution also appears different from that in New World cases: whereas in New World cases it mainly affects the nasal septum, lips, and palate, *L. infantum* infection in Spain frequently involved the laryngeal, nasal, and oral mucosa, with additional cases in the trachea and bronchi [43]. The lesions were often ulcerative, nodular, polypoid, or mass-like, sometimes closely mimicking malignancy [44].

In contrast to New World mucocutaneous leishmaniasis, for which treatment is relatively better defined and usually relies on systemic pentavalent antimonials, sometimes combined with pentoxifylline, with amphotericin B mainly reserved for selected situations [5], treatment of autochthonous mucocutaneous leishmaniasis due to *L. infantum* in Spain remains poorly standardised. In the Spanish cases reviewed, management was markedly heterogeneous, including liposomal amphotericin B, amphotericin B lipid formulations, meglumine antimoniate or sodium stibogluconate, miltefosine, pentamidine, azole-based rescue regimens, and occasionally surgery or excision as adjunctive measures. Several patients required treatment changes because of toxicity, persistence, or relapse, and some recent airway cases even received prolonged or maintenance liposomal amphotericin B. This variability probably reflects the rarity of the disease, the absence of specific guidance for Old World *L. infantum* mucocutaneous disease, and the clinical complexity of many patients, particularly those with immunosuppression or extensive upper airway involvement.

In New World LMC, reported risk factors include male sex, incomplete or missed antimonial treatment, large or multiple cutaneous lesions, particularly those located above the waist, specific genetic backgrounds such as HLA-DR2 and HLA-DQw3, and malnutrition. In contrast, immunosuppression does not appear to play as prominent a role [45].

Diagnosis requires definitive microbiological confirmation and, ideally, species identification, usually through culture and/or molecular methods. As these lesions may mimic neoplastic disease in both anatomical location and clinical appearance, some patients undergo aggressive initial management, including extensive surgical resection, before the correct diagnosis is established. A high index of suspicion is therefore essential. Early consideration of mucocutaneous leishmaniasis may facilitate less invasive diagnostic approaches, such as fine-needle aspiration, and enable timely curative treatment while avoiding unnecessary morbidity. The absence of preceding cutaneous lesions, together with underreporting and limited clinical awareness, likely contributes to diagnostic delay, which remains one of the main challenges in these cases.

There is broad agreement that systemic therapy is required, as local treatment alone appears insufficient. As noted above, liposomal amphotericin B is the most frequently used agent in the literature and appears particularly effective for mucocutaneous involvement caused by *Leishmania infantum*, supporting its role as a first-line option. However, the optimal regimen remains uncertain, and higher cumulative doses, similar to those used in immunosuppressed patients, may be warranted, potentially up to 40 mg/kg. Following the large leishmaniasis outbreak declared in Fuenlabrada, Madrid, in 2009, which resulted in more than 700 notified cases by December 2016, local treatment recommendations for mucocutaneous *L. infantum* disease were developed. Adapted from New World guidelines and local clinical experience, these recommendations propose intravenous liposomal amphotericin B as first-line therapy at a cumulative dose of 21–40 mg/kg, oral miltefosine as second-line therapy at 50 mg three times daily for 28 days, and pentavalent antimonials as third-line therapy at 20 mg Sb^5+^/kg administered intramuscularly or intravenously for 20 days [46].

The therapeutic recommendations presented in this review should be interpreted in the context of the very low certainty of the available evidence. To date, no randomized clinical trials or comparative studies have specifically evaluated treatment strategies for autochthonous mucocutaneous leishmaniasis caused by *Leishmania infantum*, and current management is therefore largely based on expert opinion, published case reports and case series, and extrapolation from other forms of leishmaniasis. Consequently, the recommendations reflected in this review are intended to provide a pragmatic synthesis of the best evidence currently available rather than definitive guidance. The absence of robust evidence, together with the lack of specific national or international clinical practice guidelines, likely contributes to the considerable heterogeneity observed in therapeutic approaches. Recognising these limitations is, in itself, one of the objectives of this review, as defining this entity more clearly represents a necessary first step towards improving epidemiological surveillance, facilitating collaborative research, and ultimately generating the evidence required to develop robust, evidence-based therapeutic recommendations.

Based on the available evidence, surgery should not be considered a definitive therapeutic option for mucocutaneous leishmaniasis caused by *Leishmania infantum*. Current national and international recommendations do not support surgical excision as curative treatment for this clinical presentation. Instead, the primary role of surgery is diagnostic, providing adequate tissue for histopathological evaluation and microbiological confirmation. Given that mucosal involvement reflects an infectious process rather than a purely localized lesion, definitive management should rely on systemic antiparasitic therapy.

Recognition of this entity in our setting is essential, and Spain should be acknowledged as an endemic area for mucocutaneous involvement caused by *Leishmania infantum*. It is imperative that official bodies compile and evaluate the accumulated evidence to establish standardised treatment guidelines. Such guidance would help reduce the current heterogeneity in therapeutic approaches and support more consistent, evidence-based clinical management.

This review does not include all reported cases of mucocutaneous involvement caused by *Leishmania infantum*. Cases lacking definitive species identification were excluded, even when the epidemiological context strongly suggested *L. infantum* as the causative agent.

Restricting the analysis to microbiologically confirmed *Leishmania infantum* cases provides a robust framework for describing the epidemiological and clinical characteristics of autochthonous mucocutaneous disease attributable to this species. At the same time, it is likely that additional cases have remained unconfirmed at the species level and were therefore not captured in this review. Rather than weakening the findings, this reinforces the notion that mucosal involvement caused by *L. infantum* is probably more frequent than currently recognised and remains underdiagnosed and underreported in the literature. Consequently, the present review should be interpreted as a comprehensive characterisation of confirmed cases rather than as an estimate of the true burden of this clinical entity.

Despite only a limited number of microbiologically confirmed cases fulfilling the inclusion criteria, these cases span several decades and originate from multiple geographically distinct regions of Spain, without identifiable epidemiological links between them. This temporal and geographical consistency supports the presence of an established autochthonous transmission cycle capable of producing mucocutaneous disease due to *Leishmania infantum*, rather than representing isolated or anecdotal events. While this clinical presentation is clearly much less frequent than visceral or cutaneous leishmaniasis, its repeated occurrence over time suggests that it constitutes a genuine and reproducible manifestation of autochthonous *L. infantum* infection. Therefore, the rarity of reported cases should not be interpreted as evidence that this entity is exceptional, but rather as a reflection of its probable underrecognition, underdiagnosis, and underreporting.

As with any review of published cases, publication and reporting bias may have influenced the available evidence, since unusual, severe, or clinically distinctive presentations are more likely to be reported than typical or uncomplicated cases. Consequently, the frequency and clinical spectrum described in this review should be interpreted with appropriate caution.

## 5. Conclusions

Mucocutaneous involvement by *Leishmania infantum* represents a distinct, underdiagnosed clinical manifestation, different from both cutaneous and visceral leishmaniasis. Its limited recognition contributes to low clinical suspicion and to the misconception that Spain is not an endemic area for mucocutaneous disease caused by this species. It mainly affects men, particularly those with immunosuppression, and likely reflects parasite dissemination beyond the inoculation site. Systemic treatment is required, with liposomal amphotericin B as the preferred first-line option, while miltefosine and pentavalent antimonials remain alternative therapies.

## 6. Future Directions

Recognition of this entity in official guidelines and the development of standardised treatment protocols are needed to reduce current therapeutic heterogeneity. Strengthening the notification and systematic reporting of MCL cases in Spain is a critical step toward improving epidemiological surveillance and ensuring the inclusion of Spain as an endemic country for this entity in official WHO records. Increased recognition of autochthonous MCL as an established clinical entity in our setting is already contributing to greater clinical awareness, earlier diagnostic suspicion, and a reduction in diagnostic delay. In this context, collaborative initiatives such as the Red Nacional de Leishmania (RENLeish), currently composed of 49 collaborating centers throughout Spain, are playing a central role in improving disease characterization and nationwide surveillance. This initiative was established to complement the current Spanish National Epidemiological Surveillance Network (RENAVE), which, although essential for epidemiological surveillance, does not routinely collect detailed clinical information such as patients’ immunological status, therapeutic management, or clinical outcomes. By systematically gathering these data, RENLeish aims to strengthen the clinical surveillance of leishmaniasis and provide a more comprehensive understanding of its epidemiology and management. By centralizing expertise and reinforcing accurate case notification, these networks are generating the evidence required to develop specific diagnostic and therapeutic guidelines for autochthonous *L. infantum* MCL, ultimately enabling a more standardized and evidence-based approach to the management of this entity in our country.

## Data Availability

The original contributions presented in this study are included in the article. Further inquiries can be directed to the corresponding author.
